# Microsporidia Are Natural Intracellular Parasites of the Nematode Caenorhabditis elegans

**DOI:** 10.1371/journal.pbio.0060309

**Published:** 2008-12-09

**Authors:** Emily R Troemel, Marie-Anne Félix, Noah K Whiteman, Antoine Barrière, Frederick M Ausubel

**Affiliations:** 1 Department of Genetics, Harvard Medical School, Department of Molecular Biology and Center for Computational and Integrative Biology, Massachusetts General Hospital, Boston, Massachusetts, United States of America; 2 Institut Jacques Monod, Centre National de la Recherche Scientifique, Universities Paris 6 and 7, Paris, France; 3 Department of Organismic and Evolutionary Biology, Harvard University, Cambridge, Massachusetts, United States of America; University of Vermont, United States Of America

## Abstract

For decades the soil nematode Caenorhabditis elegans has been an important model system for biology, but little is known about its natural ecology. Recently, C. elegans has become the focus of studies of innate immunity and several pathogens have been shown to cause lethal intestinal infections in *C. elegans.* However none of these pathogens has been shown to invade nematode intestinal cells, and no pathogen has been isolated from wild-caught C. elegans. Here we describe an intracellular pathogen isolated from wild-caught C. elegans that we show is a new species of microsporidia. Microsporidia comprise a large class of eukaryotic intracellular parasites that are medically and agriculturally important, but poorly understood. We show that microsporidian infection of the C. elegans intestine proceeds through distinct stages and is transmitted horizontally. Disruption of a conserved cytoskeletal structure in the intestine called the terminal web correlates with the release of microsporidian spores from infected cells, and appears to be part of a novel mechanism by which intracellular pathogens exit from infected cells. Unlike in bacterial intestinal infections, the p38 MAPK and insulin/insulin-like growth factor (IGF) signaling pathways do not appear to play substantial roles in resistance to microsporidian infection in C. elegans. We found microsporidia in multiple wild-caught isolates of *Caenorhabditis* nematodes from diverse geographic locations. These results indicate that microsporidia are common parasites of C. elegans in the wild. In addition, the interaction between C. elegans and its natural microsporidian parasites provides a system in which to dissect intracellular intestinal infection in vivo and insight into the diversity of pathogenic mechanisms used by intracellular microbes.

## Author Summary

The small roundworm Caenorhabditis elegans is an important model system for many areas of biology, but little is known about its natural ecology. We have identified an intracellular parasite from C. elegans in its natural habitat isolated near Paris and have named it Nematocida parisii, or nematode-killer from Paris. N. parisii defines a new genus and species of microsporidia. Microsporidia are ubiquitous eukaryotic pathogens that are thought to be highly reduced fungi and are emerging pathogens of humans. The microsporidian N. parisii invades and resides in C. elegans intestinal cells where it goes through a multistep life cycle and eventually escapes out of intestinal cells, leaving holes in the terminal web, an important cellular structure. We have found N. parisii and a related *Nematocida* species in several wild-caught roundworms, indicating that microsporidian infections may be relatively common for C. elegans in the wild. The *C. elegans/N. parisii* interaction provides a valuable system in which to study microsporidian infections in a whole animal, and a convenient and inexpensive system in which to screen for anti-microsporidian drugs.

## Abstract 3

A newly identified intracellular pathogen of wild-caught*Caenorhabditis elegans* represents a new microsporidian species that will provide a model to study this class of pathogen in humans.

## Introduction

The intestine is a major route for pathogens to invade the body. Pathogens have evolved sophisticated mechanisms to exploit the host cell machinery of intestinal cells in order to survive and replicate in this environment [1–4]. For example, the bacterium Listeria monocytogenes uses the mammalian endocytic pathway to invade intestinal epithelial cells and then induces actin polymerization, which propels the invading bacteria both within and between host cells [5]. However, many questions remain about how pathogens interact with the intestine in vivo. For convenience, studies of intracellular pathogens of the intestine are often performed in tissue culture cells, which lack the characteristic features of intact intestinal cells. In vivo, intestinal epithelial cells are polarized and contain a “brush border” on their apical side. The brush border is decorated with finger-like microvilli, which are anchored by a conserved cytoskeletal structure called the terminal web. Little is known about what role these differentiated features play during the infection process or how they are manipulated by pathogens.

The nematode C. elegans has become an attractive model for exploring host/pathogen interactions in the intestine. In its natural environment, C. elegans feeds on a variety of microbes. C. elegans does not appear to have professional immune cells, and therefore relies mainly on epithelial immunity to fight off microbial infections in the intestine. Several different microbial pathogens have been shown to infect and kill C. elegans in the laboratory and defense against these pathogens involves conserved innate immune signaling pathways [6,7]. Many pathogens infect the C. elegans intestine, which is composed of only 20 large epithelial cells that are easily visible because C. elegans is transparent [8]. These epithelial cells contain apical microvilli anchored into a terminal web of actin and intermediate filaments. Such morphological features are characteristic of intestinal cells in mammals, thus making C. elegans an excellent model for understanding interactions between enteric pathogens and intestinal cells. No pathogens have so far been described that reside intracellularly in C. elegans intestinal cells, even though several intracellular pathogens of mammals have been shown to establish lethal intestinal infections [9,10]. The failure of human intracellular intestinal pathogens to invade C. elegans intestinal cells is most likely a consequence of the fact that these pathogens are not natural pathogens of C. elegans. Little is known about the natural pathogens of C. elegans and so far, no pathogen has been isolated from wild-caught C. elegans individuals.

To learn more about the ecological pressures on C. elegans and to develop an accessible in vivo model for intracellular infection of the intestine, we characterized a natural intracellular pathogen of C. elegans. We show here that this pathogen is a novel species of microsporidia. Microsporidia are obligate intracellular eukaryotic parasites most closely related to the fungi [11,12]. They infect a wide range of hosts, including vertebrate and invertebrate animals, as well as some protists. Microsporidia commonly infect insects, and have agricultural significance both as a parasite of honeybees, but also as a biocontrol agent for insect pests. There has been a recent surge of medical interest in microsporidia*.* At least 15 different microsporidian species have been shown to infect humans, and microsporidia have recently been added to the National Institutes of Health list of priority pathogens, as well as the Environmental Protection Agency list of waterborne microbial contaminants of concern [13]. Microsporidia frequently infect the intestine, which can cause self-limiting diarrhea in immunocompetent patients, but can lead to severe, persistent diarrhea in immunocompromised patients [14].

Microsporidia survive outside of their hosts as spores. Microsporidian spores are distinguished by a striking infection apparatus called the polar tube, which everts to pierce the membrane of the host cell [15]. The polar tube then acts as a syringe to directly inject nuclei and sporoplasm into the host. Once inside the host cell, microsporidia replicate in a cell-wall deficient form called a meront, which eventually differentiates to re-generate the spore form. Despite the medical and agricultural relevance of these parasites, little is known about the pathogenic mechanisms used by these ubiquitous microbes, including how microsporidian spores exit intestinal cells to go on to infect new hosts.

In our study of a natural intracellular infection of the C. elegans intestine, we have discovered a new microsporidian species that we have named Nematocida parisii, which defines a new genus and a new species. N. parisii is transmitted horizontally, i.e., from animal to animal. Infection with N. parisii proceeds through a distinct series of stages within the intestinal cell, starting with a meront stage, in which irregularly shaped microbes called meronts cause “grooves” in the intestine. Later in the infection when these meronts develop into spores, striking gaps appear in the terminal web underlying the intestinal microvilli. These changes occur when animals become infectious to others, so this may be part of an exit strategy for infectious microsporidian spores. Defense against microsporidian infection does not appear to involve the p38 mitogen-activated protein kinase (MAPK) or insulin signaling pathways, which are involved in defense against a variety of bacterial and fungal pathogens in *C. elegans.* In addition to the original microsporidian isolate we characterized, we have also found microsporidia infections in several other natural isolates of *Caenorhabditis* nematodes from diverse geographical locations. The *C. elegans/N. parisii* model provides a new system for exploring the specific mechanisms of microsporidia pathogenicity, as well as the more general strategies used by intracellular microbes to survive and replicate in the intestine of animals.

## Results

### An Intracellular Microbe From a Wild-Caught C. elegans Is Transmitted Horizontally

**Figure 1:**
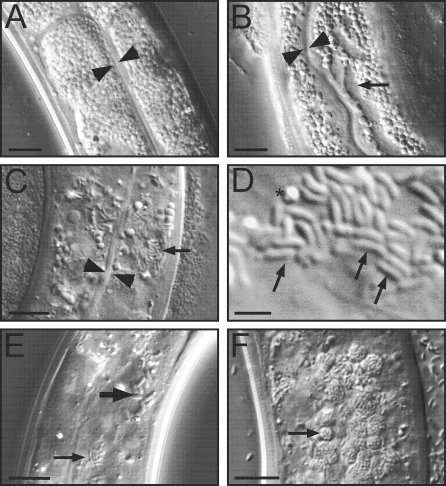
Intracellular Infection of C. elegans Proceeds through Distinct Stages (A) Uninfected intestine. (B) Infection causes displacement of gut granules, which appear as “grooves,” indicated by arrow. (C) Small rod-shaped microbes in intestine, indicated by arrow. Arrowheads indicate intestinal lumen in (A–C). (D) Higher magnification view of rod-shaped microbes. Asterisk marks gut granule. (E) Large and small rod-shaped microbes are indicated by larger and smaller arrow, respectively. (F) Vesicles of microbes are indicated by arrow. Scale bar is 10 μm in (A–C), (E), (F), and 2 μm in (D).

A wild-caught C. elegans strain isolated from a compost pit in Franconville, France (near Paris) was found to harbor small, rod-shaped microbes in its intestinal cells (compare Figure 1C–1F to uninfected animals in Figure 1A) [16]. These microbes are transmitted horizontally at very high efficiency from animal to animal: if donor “infected” animals are incubated on the same plate as uninfected “recipient” animals, 100% of the recipient animals become infected with the rod-shaped microbes (*n* > 30 donors tested, for each donor, 20 recipients were examined). Because the infection is readily transferred to the standard N2 laboratory strain of *C. elegans,* further characterization has been performed in this strain background. The infection does not appear to be transmitted vertically, because noninfected progeny could be isolated from their hermaphrodite infected parents in two different ways. First, eggs could be isolated from infected parents by bleaching gravid adults, and these eggs developed into uninfected adults (*n* = 263 animals examined). Second, manually separating progeny from their infected parents just before or immediately after hatching allowed them to develop into uninfected adults (*n* = 27 animals examined).

Because we have been unable to culture an infecting microbe(s) outside of the host (see next section), we generate infectious extracts that can be used to reproducibly transmit the infection. Infected animals are mechanically disrupted, filtered, and then frozen as a glycerol stock (see Materials and Methods for details). Using these extracts, all C. elegans postembryonic developmental stages have been shown to be susceptible to infection, except for dauer larvae, which do not feed.

The first sign of infection appears within a day or two: distinct regions devoid of gut granules appear in the intestinal cells. These initial symptoms can arise at any point within the intestine. Early in infection, these regions are small circles, and later extend to longer grooves, as shown in Figure 1B. After the appearance of these grooves, rod-shaped microbes become visible (2.18 ± 0.15 μm long, 0.8 ± 0.08 μm wide, *n* = 47, Figure 1C–1E). Subsequently, slightly larger rod-shaped microbes are sometimes also observed (3.17 ± 0.22 μm long, 1.31 ± 0.15 μm wide, *n* = 41, Figure 1E). When the intestine becomes heavily infected with microbes, the rod-shaped microbes are found in discrete vesicles, as shown in Figure 1F. Eventually all infected worms die prematurely compared to noninfected controls (see Figure 5A). While the exact cause of death is difficult to determine, one likely cause of death is that intestinal cells become completely filled with rod-shaped microbes and thus are no longer able to absorb and transmit nutrients.

When animals are initially infected with rod-shaped microbes, they appear grossly normal by examination with a dissecting microscope. At 48 h postinoculation, animals with rod-shaped microbes in their intestines had normal feeding rates based on a pharyngeal pumping assay: uninfected animals pumped at 250 ± 70 pumps/minute (*n* = 21), while infected animals pumped at 247 ± 60 pumps/minute (*n* = 18). Later in the infection animals became more sluggish when their entire intestine became filled with rod-shaped microbes (e.g., Figure 1F). Infection occurred almost exclusively in the intestine, except in rare cases where heavily infected worms also had microbes in the anal region.

### Intracellular Microbe Is a New Species of Microsporidia

To determine the identity of the intracellular microbes, we performed PCR on lysate from infected animals using universal rDNA primers. Infected animals were rinsed, lysed, and then used as template for PCR. The resulting PCR product was cloned, sequenced, and analyzed by BLAST. Using primers that hybridize to prokaryotic rDNA, we found sequence corresponding to two species of Gram-negative bacteria: Pseudomonas putida and a previously unknown *Ochrobactrum* strain, both of which could be cultured on defined media (see Materials and Methods). However, following repeated treatment with antibiotics (kanamycin, tetracycline, and gentamicin), a strain of animals was obtained that still contained a highly infectious intracellular pathogen, but no longer contained any detectable bacteria, as determined by culturing or by PCR (see Figure S1 and Text S1 for more information on *Ochrobactrum*).

**Figure 2:**
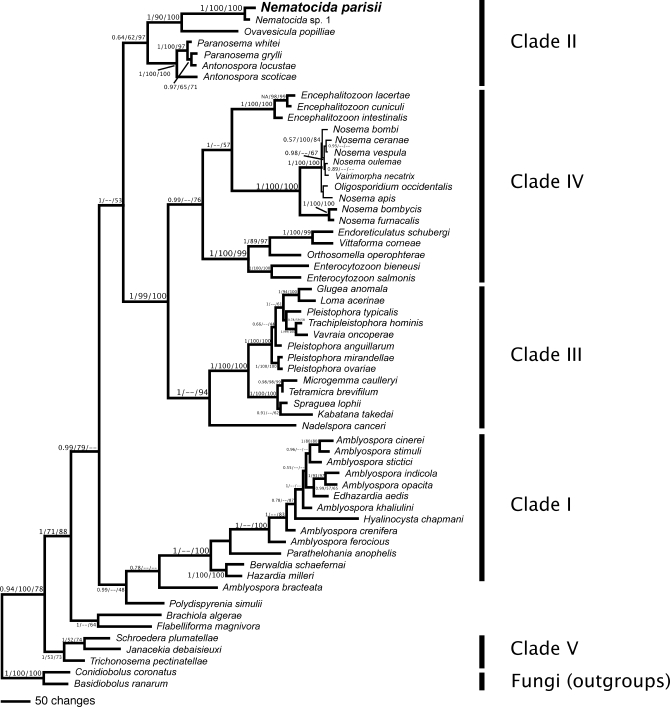
Phylogenetic Analysis of Microsporidian Sequence Isolated from Infected Nematodes Bayesian inference phylogeny of the small subunit ribosomal DNA from selected microsporidia and close relatives, estimated using the program MrBayes. Two fungi were used as outgroups in the analysis (see [29]). Numbers above branches are Bayesian posterior probabilities/parsimony bootstrap/maximum likelihood bootstrap values. If nodal support values were below 0.5 or below 50% for any analysis we indicated this by “—“. The five major clades of Microsporidia are indicated on the right of the figure (see [29]). Note the relationship between *Nematocida* and O. popilliae.

When PCR was performed on lysate from infected animals using primers that hybridize to both prokaryotic and eukaryotic rDNA, sequence was obtained that corresponds to the phylum Microsporidia, which comprises eukaryotic intracellular pathogens. This rDNA sequence is most closely related to rDNA sequence from Ovavesicula popilliae, which is a beetle-infecting microsporidian species [17], although the two sequences are fairly divergent (see phylogenetic comparison in Figure 2). Because of this divergence and the fact that the putative microsporidian species infecting C. elegans is also morphologically distinct (O. popilliae spores are larger and oval-shaped) we gave the C. elegans microbe a new genus and species name: Nematocida parisii for nematode killer from Paris (see Taxonomic Summary for the generic and species descriptions).

**Figure 3:**
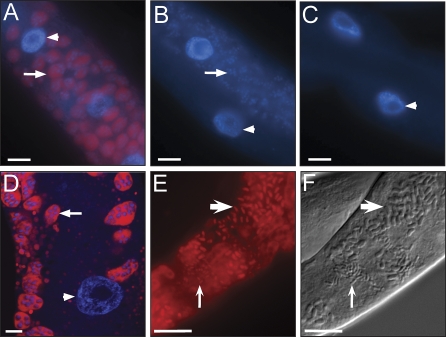
Intracellular Microbe Is a Novel Microsporidia Species (A–E) FISH staining of C. elegans intestines using Cy3 probes for rRNA in red and (A–D) counterstained with DAPI in blue to label DNA. (A) Infected animal at the groove stage stained with MicroA, a probe specific for N. parisii rRNA. Arrow indicates FISH and DAPI staining of multinucleate microbes (meronts). Similar results were obtained with MicroB, another probe specific for N. parisii (unpublished data). (B) Infected animal at the groove stage stained with universal probe for bacterial rRNA (EUB338) [67]. No FISH staining is observed, although excess DAPI staining is visible due to meronts, indicated with arrow. (C) Uninfected animal stained with N. parisii-specific probe. For (A–C) scale bar is 10 μm. (D) Confocal imaging of FISH staining with *N. parisii-*specific probe. Arrow indicates multinucleate microbes (meronts). Scale bar is 5 μm. For (A and D), arrowhead indicates host nucleus. (E) rRNA FISH staining of rod-shaped microbes (spores) with N. parisii-specific probe. (F) DIC image of spores. For (E and F), small spores indicated with smaller arrow, large spores indicated by larger arrow, and scale bar is 10 μm.

Because conditions have not been developed to culture microsporidia independently of host cells, we used RNA fluorescence in situ hybridization (FISH), which is a culture-independent method, to determine that N. parisii corresponds to the C. elegans intracellular microbes. As shown in Figure 3A, oligonucleotide FISH probes for N. parisii rRNA showed strong, specific staining in the intestinal grooves of infected animals, corresponding to the stage of infection shown in Figure 1B. This staining indicated that the intracellular microbe corresponds to a microsporidian species. Moreover, 4′,6-diamidino-2-phenylindole (DAPI) counterstaining to label DNA often revealed several spots within one FISH-staining cluster in the grooves, indicating these were multinucleate meronts (Figure 3D). As controls, a universal bacterial probe for rRNA did not label infected worms (Figure 3B), nor did the N. parisii-specific probes label intestinal cells of uninfected worms (Figure 3C).

After microsporidian meronts replicate, they form a thick cell wall and differentiate into the spore form, which is often oval or rod-shaped. FISH staining of infected animals (corresponding to the stage of infection shown in Figure 1E) resulted in labeling of the rod-shaped structures, both small and large (Figure 3E and 3F), which likely correspond to N. parisii spores. We refer to microbes in the groove stage as “meronts” and microbes in the rod-shaped stage as “spores” by analogy with other microsporidian species.

**Figure 4:**
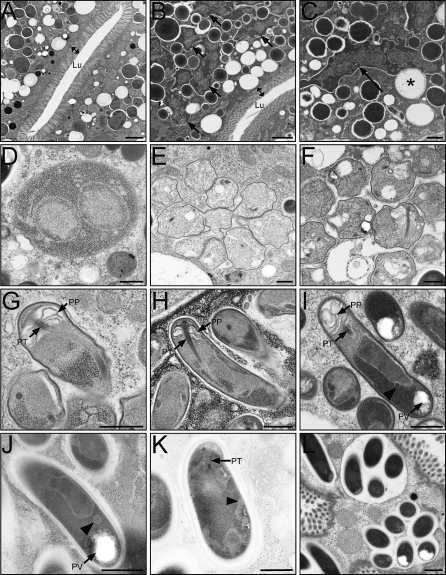
Transmission Electron Micrographs of N. parisii Infection in the C. elegans Intestine (A) Uninfected adult intestine. (B) Adult intestine 30 h postinoculation (at groove stage by light microscopy). N. parisii meronts are indicated with arrows. In (A and B), double arrows indicate microvilli and Lu indicates lumen. Scale bar is 2 μm. (C) Higher magnification view of multinucleate meront indicated with an arrow in close proximity to host vesicle marked with an asterisk. Scale bar is 1 μm. (D) Later stage meront with two nuclei and more regularly shaped plasma membrane, 44 h postinoculation. (E and F) Adult intestine 44 h postinoculation with stages intermediate between meront and spore. (G–I) Adult intestine 44 h postinoculation containing developing N. parisii spores. (J) Mature spore. (K) Larger sized spore (comparable to larger microbes in Figure 1E). For (G–K) PT refers to the anterior portion of the polar tube called the manubroid [68], PP refers to the polaroplast membranes, PV refers to the posterior vacuole and arrowheads indicate cross-sections of polar tube coils. (L) Vesicles surrounding spores (comparable to vesicles in Figure 1F). For (D–L), scale bar is 500 nm.

Transmission electron microscopy (TEM) analysis was used to more closely examine N. parisii at different stages of infection. During the groove stage of infection multinucleate meronts are visible as irregularly shaped structures (compare Figure 4B and 4C to uninfected animal in Figure 4A). In several cases, meronts appear to be attached to intracellular vesicles: of 124 meronts examined at 30 to 32 h postinoculation, 48% have curvature around at least one-quarter of the circumference of an intracellular vesicle (six sections examined from two different experiments). While it is difficult to assign identity to these vesicles without immunostaining, one possibility is that they contain nutrients that the microsporidia are able to absorb (note the lack of distinct membrane between meront and host vesicle in Figure 4C). Later in the infection, multinuclear structures with more regularly shaped membranes are observed, which are also likely meronts (Figure 4D). In both the early meront form (Figure 4C) and the late meront form (Figure 4D), meronts could contain one or multiple nuclei, which did not appear to be paired, suggesting this species is monokaryotic.

**Figure 5:**
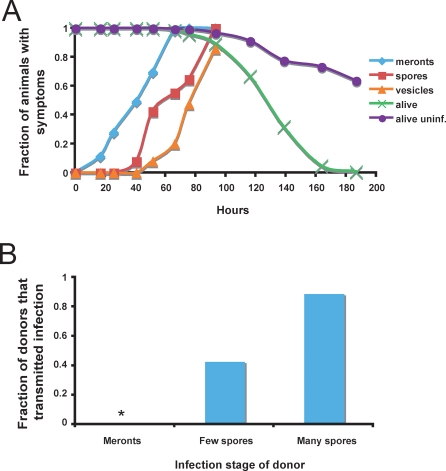
Timecourse of N. parisii Infection and Transmission of Infection (A) Progression of infection in wild-type animals. L1s were infected with N. parisii at 0 h, and 80–150 animals were scored at each timepoint for meronts, spores, or vesicles by DIC microscopy, and for survival by prodding animals with a platinum wire. “Alive uninf.” refers to survival of uninfected animals grown in parallel with infected animals. The time to 50% of animals exhibiting symptoms was 43.1 ± 3.7 h for meronts, 65.8 h for spores, and 75.9 ± 1.6 h for vesicles. The time to 50% animals dead (TD50) was 127 ± 2 h. There was a statistically significant difference between the survival of infected and uninfected animals from 116 h onward (116 h, *p* = .02; 138.5 h, *p* = .0057; 163.5 h, *p* = .00015; 186.5 h, *p* = .00011). See Materials and Methods for analysis details. (B) Transmission of infection. Fraction of infected donor animals that transmitted infection is indicated on *y*-axis. None of the 16 donors infected only with meronts transmitted the infection (asterisk indicates fraction equals 0). 16 of 18 donors with many spores (>100 spores) transmitted the infection. Three of seven donors with only a few spores (<100 spores) transmitted the infection. See Materials and Methods for assay details.

Later in the infection, a variety of microbe shapes are observed. These include microbes that have shapes intermediate between meronts and spores, which are likely to be sporoblasts (precursors to spores) (Figure 4E and 4F). Also visible are microbes that appear to be more differentiated spores (Figure 4G–4L). As microbes differentiate into spores, a structure likely to be the polar tube infection apparatus becomes visible (Figures 4G–4I). Also visible are lamellar membranes that likely are the polaroplast membranes, in which the polar tube is positioned. Polar tubes in many microsporidian species are so long that they wrap around several times near the posterior end of the spore and are visible as coils in cross-section. In smaller sized N. parisii spores, one cross-section of the polar tube coil was sometimes observed near the posterior end, often at the interface of a punctate region (perhaps containing ribosomes) and a more uniform region (e.g., Figure 4I). Smaller-sized spores as shown in Figure 4I, 4J, and 4L averaged 1.89 ± 0.24 μm long and 0.54 ± 0.07 μm wide, as measured by TEM (*n* = 65). Larger-sized spores as shown in Figure 4K had as many as five polar tube coils visible, often near the edge of the spore. Posterior vacuoles were sometimes observed in spores as well (Figure 4I and 4J). In general, these morphological characteristics are considered diagnostic for identification of microsporidian infections [11]. Also observed were membranes forming around clusters of mature spores (Figure 4L).

### Characterization of the N. parisii Infection Cycle

To precisely measure the kinetics of infection we inoculated a staged population of wild-type animals with infectious extract and then examined for different stages of infection over several days by differential interference contrast (DIC) microscopy. A typical time course is illustrated in Figure 5A. Meronts appear within the first day, and spores are visible a day later, followed by vesicles filled with spores. Animals begin dying around the fourth day of infection. In many assays, a biphasic curve is observed for spore infection. The first part of the curve (before about 60 h) may reflect animals infected by the initial infectious dose, with the second phase (after about 60 h) reflecting animals being infected by their contagious neighbors.

Next we determined when the infection was transmitted from donor animals. “Donor” infected animals were incubated on the same plate as “recipient” uninfected animals for 12 h, then donor animals were removed from the plate and examined by DIC microscopy for the presence of meronts or spores. All donor animals were alive and active when removed from the plate and their intestinal cells appeared intact by light microscopy. Two days later recipients were examined to determine whether they had received the infection (Figure 5B). We found that animals infected only with meronts were not infectious to others. In contrast, a majority of donor animals with spores transmitted the infection, including some animals that only had a small number of spores visible. The fact that animals with only a small number of spores could transmit the infection suggests that infectious spores can exit intestinal cells without causing much damage, since donor animals had grossly normal intestinal cells as assessed by light microscopy. We also found that animals containing only the small-sized spores could transmit the infection to others and this transmission resulted in recipients that were infected with both small and large-sized spores (*n* = 3 donors).

### N. parisii Infection Causes Gaps in the Terminal Web Underlying Intestinal Microvilli

**Figure 6:**
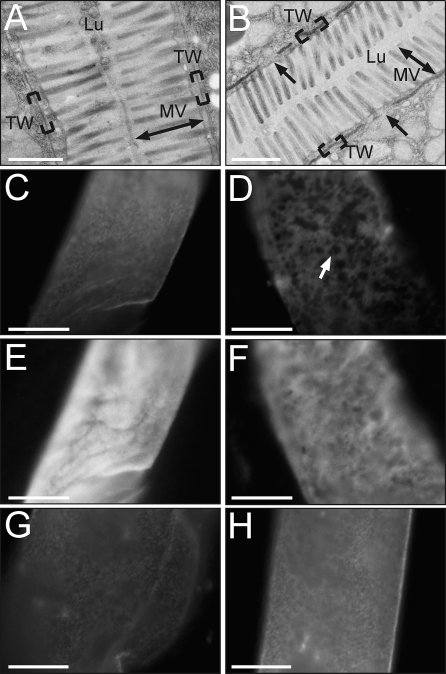
Infected Animals Have Lesions in the Terminal Web (A) Electron micrograph showing cross-section of intestine in uninfected animal. (B) Micrograph showing cross-section of intestine in N. parisii infected animal. Gaps in terminal web are indicated with arrows. (A, B) Brackets indicate terminal web (TW), double arrow indicates microvilli (MV), Lu indicates lumen, scale bar is 1 μm. (C) Uninfected intestine stained with MH33 antibody, which labels IFB-2, an intermediate filament component in the terminal web. Terminal web staining appears as a sheet, since image is from nonconfocal microscope. (D) Infected intestine stained with MH33. Gaps in MH33 staining are indicated with an arrow. (E) Same uninfected intestine as in (C), but visualizing ERM-1::GFP. (F) Same infected intestine as in (D), but visualizing ERM-1::GFP. Animal stained with MH33 antibody after being infected with P. aeruginosa strain PA14 for 27 h (G) or S. aureus strain NCTC 8325 for 24 h (H). Note gut distension due to PA14 infection. Scale bar is 10 μm (C–H).

To investigate how spores escape from intestinal cells, we examined structural features of intestinal cells infected with spores. Electron microscopic analysis of infected intestines indicated that N. parisii infection causes specific damage to the terminal web, a cytoskeletal structure found in many polarized epithelial cells. The terminal web is thought to be composed of actin and intermediate filaments that provide structural support for the actin-rich microvilli [18]. A normal terminal web in C. elegans intestinal cells can be seen as an electron-dense structure underlying the finger-like microvilli, as shown in Figure 6A. In electron micrographs from N. parisii infected animals, parts of the terminal web appear to be missing (Figure 6B), although the microvilli appear largely intact, and cells contain normal-looking apical junctions (unpublished data), which are cytoskeletal structures used to connect neighboring intestinal cells.

To further examine this apparent structural damage to the terminal web, immunofluorescence was performed on infected worms to label components of the terminal web with specific molecular markers. The monoclonal antibody MH33 specifically labels the intermediate filament protein IFB-2, which is a major component of the terminal web in C. elegans intestinal cells [18]. IFB-2 immunostaining in animals infected with spores revealed gaps in the normally continuous sheet corresponding to the terminal web (compare Figure 6C and 6D). To determine whether other cytoskeletal structures are damaged in infected cells, we examined the cytoskeleton membrane linker protein, ERM-1, which is localized to the apical side of intestinal cells [19], likely in the microvilli. Visualizing an ERM-1::green fluorescent protein (GFP) translational fusion in animals that were also immunostained against IFB-2 (Figure 6E and 6F) showed that ERM-1::GFP had occasional gaps in staining, but these were much less consistent and less dramatic than the gaps in IFB-2 immunostaining. These results suggest that N. parisii infection causes more damage to intermediate filaments and the terminal web than to microvilli, consistent with electron micrographs. Because these intermediate filament gaps occur in animals infected with spores, which is the infectious stage, these gaps may be an exit strategy for N. parisii spores.

To rule out the possibility that the observed gaps in the terminal web in N. parisii infected animals are caused by nonspecific damage to the intestine, we examined IFB-2 immunostaining in animals infected with other intestinal pathogens. The Gram-negative bacterial pathogen P. aeruginosa and the Gram-positive bacterial pathogen Staphylococcus aureus kill C. elegans rapidly via intestinal infection (time to 50% animals dead [TD50] approximately 50 h) [20,21]. IFB-2 immunostaining was continuous in P. aeruginosa infected animals (Figure 6G) and S. aureus infected animals (Figure 6H) even in those that were near death. Therefore, gaps in the terminal web appear to be a specific consequence of N. parisii infection, and not the result of nonspecific damage by intestinal pathogens in general.

### The Role of Previously Identified Immunity Pathways in Resistance to N. parisii

**Figure 7:**
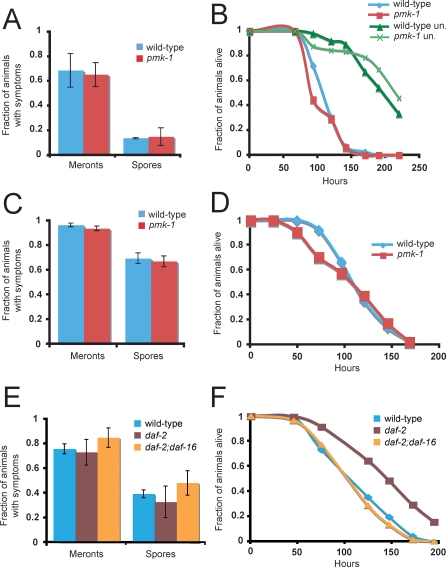
N. parisii Infection in Wild-Type and *pmk-1* Mutant Animals (A and C) Comparison of infection symptoms in wild-type and *pmk-1* mutants infected with N. parisii at the L1 stage and assayed at 43 h postinoculation (A) or infected at the L4 stage and then assayed 47 h postinoculation (C), with at least 40 animals per plate. The average of three plates is shown, error bars are standard deviation. There was not a significant difference between wild-type and *pmk-1* at the L1 stage (*p* = 0.75 for meronts, *p* = 0.39 for spores) or the L4 stage (*p* = 0.10 for meronts, *p* = 0.53 for spores). (B and D) Comparison of survival between wild-type and *pmk-1(km25)* mutants inoculated with N. parisii at L1 stage (B) or L4 stage (D). Assays were performed starting with at least 40 animals per plate, three plates per experiment. In (D), “un” refers to uninfected. There was not a significant difference between wild-type and *pmk-1* at the L1 stage (*p* = 0.0529) or the L4 stage (*p* = 0.65). (E) Comparison of infection symptoms in wild-type, *daf-2(e1368)*, and *daf-2(e1368);daf-16(mgDf47)* mutants infected with N. parisii at the L4 stage and assayed at 45 h postinoculation. The average of three plates is shown, error bars are standard deviation. There was not a significant difference between wild-type and *daf-2* (*p* = 0.69 for meronts, *p* = 0.44 for spores), between wild-type and *daf-2;daf-16* (*p* = 0.15 for meronts, *p* = 0.22 for spores) or between *daf-2* and *daf-2;daf-16* (*p* = 0.19 for meronts, *p* = 0.17 for spores). (F) Comparison of survival between wild-type, *daf-2(e1368),* and *daf-2(e1368);daf-16(mgDf47)* mutants infected with N. parisii at the L4 stage. Assays were performed starting with at least 40 animals per plate, three plates per experiment. There was a significant difference between survival of wild-type and *daf-2* (*p* < 0.0001) and between *daf-2* and *daf-2;daf-16* (*p* < 0.0001), but not between wild-type and *daf-2;daf-16* (*p* = 0.40). All data shown in the figure are representative of at least three independent experiments.

The C. elegans p38 MAPK mutant *pmk-1* is significantly more sensitive to killing than wild-type worms by most bacterial and fungal pathogens tested to date [21–24]. However, *pmk-1* mutants were not found to be infected more rapidly by N. parisii as assessed by microscopy, either when infected as first stage (L1) larvae (Figure 7A) or as fourth stage (L4) larvae (Figure 7C). *pmk-1* mutants appeared to be modestly more susceptible to killing by N. parisii early in infection (Figure 7B and 7D). This slight sensitivity occurs at a time when *pmk-1* mutants die slightly more quickly than wild-type animals in the absence of infection (Figure 7B), suggesting the slight sensitivity is not specific to N. parisii. This subtle phenotype is distinct from the robust immunocompromised phenotype seen with *pmk-1* mutants when infected with a variety of other pathogens [21–24].

The *daf-2/daf-16* insulin/insulin-like growth factor (IGF) signaling pathway regulates resistance to a variety of pathogens in C. elegans [25] and appears to act in parallel to the p38 MAPK pathway [26]. Mutations in the *daf-2* insulin receptor cause animals to be resistant to many pathogens, and this enhanced resistance requires the downstream *daf-16* FOXO transcription factor. Because *daf-2* is required for normal larval development, we could not test the role of *daf-2* in early larvae, and instead examined the role of *daf-2* in older animals (L4 larvae). We found that neither *daf-2* nor *daf-2;daf-16* mutants appeared to have substantially altered resistance to N. parisii infection as assessed by microscopy (Figure 7E). *daf-2* mutants did survive somewhat longer when infected with N. parisii, and this resistance required *daf-16*, since *daf-2;daf-16* mutants did not survive longer than wild-type animals (Figure 7F). However, *daf-2* mutants live more than twice as long as wild-type animals in the absence of infection, so it is difficult to say whether this effect is due to resistance against N. parisii, or is due to a general increase in lifespan.

The data in Figure 7 indicate that the PMK-1 p38 MAPK and the DAF-2/DAF-16 pathways, which are important for defense against pathogens like P. aeruginosa and S. aureus, do not play a significant role in defense against microsporidian infection. Consistent with this conclusion, we found that many genes robustly induced by P. aeruginosa and S. aureus were not robustly induced by N. parisii (Figure S2) (J.I. Irazoqui, E.R. Troemel, F.M. Ausubel, unpublished data and [26,27])*.* In addition, *nlp-31*, which is a gene induced by the pathogenic fungus Drechmeria coniospora [28], was not induced by N. parisii (unpublished data). Similarly, a D. coniospora-induced *nlp-29::GFP* reporter was not induced by N. parisii infection (unpublished data). These results suggest that the C. elegans response to N. parisii is distinct from responses to previously studied fungal and bacterial pathogens.

### Multiple Wild-Caught *Caenorhabditis* Isolates Are Infected With Microsporidia

**Figure 8:**
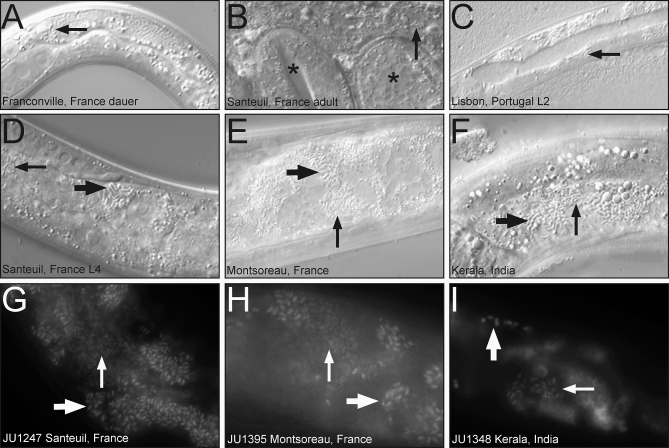
Microsporidia-Infected *Caenorhabditis* Nematodes Isolated from Diverse Geographical Locations (A) Infected dauer larva isolated in Franconville, France. (B) Infected *C. elegans* adult isolated in Santeuil, France. This adult was filled with rod-shaped microbes and unable to lay eggs. Progeny inside the adult are indicated with asterisks. (C) Infected L2 larva isolated in Lisbon, Portugal. This individual (like some of the other infected larvae) died before reaching adulthood and producing progeny. (D) Infected L4 larva isolated in Santeuil, France. This animal also died before producing progeny. DIC micrographs taken within 24 h after sampling for (A–D). (E) Infected F1 progeny of *C. elegans* isolated in Montsoreau, France. (F) Infected progeny of *C. briggsae* from India. (G–I) RNA FISH staining with MicroA, a probe for N. parisii rRNA. (G) JU1247, an infected C. elegans strain isolated in Santeuil, France. (H) JU1395, an infected C. elegans strain isolated in Montsoreau, France. (I) JU1348, an infected C. briggsae strain isolated in Kerala, India. (A–I) Smaller arrow indicates smaller rod-shaped microbes, larger arrow indicates larger rod-shaped microbes, both of which are microsporidian spores.

We found several other wild-caught nematodes from a variety of geographical locations that harbored intestinal rod-shaped microbes similar in appearance to N. parisii. Infected nematodes were isolated from multiple regions of France, from Portugal, and from India (see Materials and Methods). These infections were observed directly in individuals coming from the wild, and infected animals were found at a variety of developmental stages (Figure 8). JU1247 is a wild-caught C. elegans strain isolated from a park near Paris that is about 30 km from the original microsporidia-infected isolate (found in Franconville). JU1395 is a strain of C. elegans isolated from a compost heap in Montsoreau, France, which is 300 km south of Paris. Both of these C. elegans strains harbored intracellular infections in their intestinal cells with similar characteristics to N. parisii, i.e., they exhibited grooves and had rod-shaped microbes of two distinct sizes. rDNA fragments were isolated from infected JU1247 and JU1395 animals that exactly matched the sequence of N. parisii (unpublished data). In addition, we isolated JU1348, a strain of C. briggsae from a nature preserve in Kerala, India, which also has an intestinal microsporidian infection with characteristics similar to N. parisii (grooves and rod-shaped microbes of two distinct sizes). A fragment of rDNA was isolated from this strain that is approximately 95% identical to N. parisii rDNA. This rDNA sequence is most closely related to N. parisii in phylogenetic analysis (Figure 2), and we refer to it as Nematocida sp. 1. In order to confirm that the three *Caenorhabditis* strains mentioned above are infected with microsporidia, we performed RNA FISH using an N. parisii probe (which hybridizes to a region that is identical in both N. parisii and Nematocida sp. 1. rRNA) and saw positive signal for all three strains tested (Figure 8G–8I).

Microsporidia have been placed into five major clades, based on the phylogenetic analysis of Vossbrinck and Debrunner-Vossbrinck [29]. We have performed phylogenetic analyses using three independent methods and broadly recover these five clades from our analyses (Figure 2 and Materials and Methods). In all three analyses, the two *Nematocida* sequences (N. parisii from France and *Nematocida* sp. 1 from India) group together and share a most recent common ancestor. The *Nematocida* lineage was always placed sister to O. popilliae with high support in all three analyses. These three sequences were in turn placed sister to a group comprising Paranosema whitei, P. grylli, Antonospora locustae, and A. scoticae in all three analyses. The group comprising all of these sequences corresponds to one of two major lineages within Clade II of Vossbrinck and Debrunner-Vossbrinck [29].

### Taxonomic Summary

#### *Nematocida* defines a new genus: urn:lsid:zoobank.org:act:BE50CD2B-0F8F-4E20-BFD5-219E3BC84933.

The *Nematocida* genus comprises Microsporidia with two spore size classes that are parasites of terrestrial nematodes in the genus *Caenorhabditis*. The mode of infection is horizontal and the site of infection is the host intestine. Species form a highly distinct lineage most closely related to *Ovavesicula* in phylogenetic analysis of small subunit rDNA, which is nested in an insect-infecting lineage within Clade II of Vossbrinck and Debrunner-Vossbrinck [29]. The etymology of the generic name, “nematode killer,” denotes the virulence of the parasite, which kills its hosts. The type species is N. parisii sp. nov.

#### N. parisii defines a new species: urn:lsid:zoobank.org:act:3D12C087-94B5-4CE7-8903–71081B950061.

The type host is the nematode *Caenorhabditis elegans.* Transmission is horizontal, likely via the oral route. C. elegans containing only a small number of the small-sized spores are able to transmit the infection to neighbors. There is no evidence of vertical transmission.

#### Lifecycle and symptoms in the host.

By Nomarski light microscopy, the first sign of infection is a displacement of gut granules, causing “grooves” in the intestine. These usually occur a day after inoculation. The next stage of infection is the appearance of rod-shaped microbes (spores), which generally occur a day after the appearance of grooves. First smaller sized spores appear, and then later, larger spores as well. Eventually these spores cluster into discrete vesicles.

Morphological development of N. parisii appears to be monokaryotic in all stages examined. Two distinct meront types were observed by electron microscopic analysis. The initially observed meront type has irregularly shaped membranes and has one or several nuclei. A later-stage meront type has more regularly shaped membranes, and also has one or several nuclei. Two distinct sizes of spores are observed (see below).

The site of infection is all intestinal cells of C. elegans, which appear to be susceptible to infection. Early in the infection microbes were only seen in the intestine, while later in the infection microbes were occasionally observed in the anal region. All postembryonic stages of C. elegans were susceptible except dauer larvae, which do not feed.

There are two distinct sizes of spores visible by light microscopy, measuring 2.18 ± 0.15 μm long, 0.8 ± 0.08 μm wide and 3.17 ± 0.22 μm long, 1.31 ± 0.15 wide, respectively. One polar tube coil is occasionally visible in TEM longitudinal sections of smaller spores, while up to five polar tube coils are visible in longitudinal sections of larger spores.

The type strain was isolated from a compost pile in Franconville, Val d'Oise, Ile-de-France, France, sampled on November 24, 2004.

The etymology of the type species name N. parisii reflects the fact that it was discovered near Paris, France.

Living spores of this species have been deposited in the American Type Culture Collection (ATCC) Protistology Collection as PRA-289.

## Discussion

### N. parisii Is a Natural Intracellular Parasite of C. elegans

Here we describe a natural intracellular parasite of the nematode C. elegans, which we show is a new species of microsporidia and have named N. parisii. To our knowledge, N. parisii is the first pathogen isolated directly from a wild-caught C. elegans strain, and is the first pathogen shown to invade and reside within C. elegans intestinal cells. Our discovery of multiple natural isolates of C. elegans infected with microsporidia presents a new perspective on the challenges that C. elegans faces in its natural habitat.

**Figure 9:**
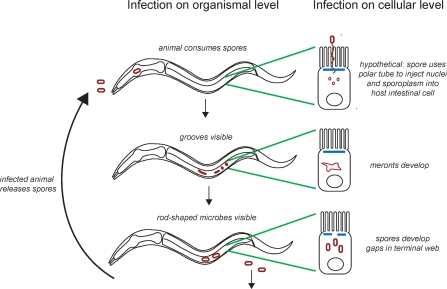
Model of N. parisii Infection in C. elegans on Cellular and Organismal Level See text for details.

The lifecycle of N. parisii infection of C. elegans is modeled in Figure 9. Once nematodes have ingested N. parisii spores, these spores likely use a polar tube infection apparatus to directly inject the intestinal host cells with microsporidia nuclei and sporoplasm. While this event has not been directly observed with N. parisii, it is quite likely to occur, because a presumptive polar tube was observed in TEM cross-sections of N. parisii spores, and since all microsporidian species are thought to use a polar tube to inject host cells with spore material. Material directly injected into host intestinal cells form multinucleate meronts, which appear in the C. elegans intestine as irregularly shaped microbes by electron microscopy and stain with a microsporidian-specific RNA FISH probe peppered with DAPI-staining nuclei. Replication of meronts displaces or consumes gut granules inside the intestinal cells, causing “grooves,” which are visible by light microscopy. These meronts eventually differentiate into spores, which show the characteristic features of microsporidia, including a polar tube. When spores have formed, gaps become visible in the terminal web. At this time, animals become infectious to others, likely through shedding of virulent spores.

We found that C. elegans p38 MAPK *pmk-1* mutants do not have substantially altered resistance to N. parisii infection, whereas *pmk-1* mutants are highly susceptible to a wide of variety of human bacterial and fungal pathogens that have been tested [21,22,24]. One possible explanation for this observation is that because of a long-term evolutionary arms race with nematodes, N. parisii has evolved mechanisms to suppress PMK-1-mediated immune responses, analogous to the suppression of basal immune responses in plants mediated by Type III secretion system effectors of highly evolved plant pathogens such as P. syringae [30–32]. Alternatively, the lack of p38 MAPK involvement in resistance against microsporidia may be due to the intracellular lifestyle of N. parisii, which could allow this pathogen to evade detection of immune receptors or evade the activity of antimicrobials controlled by the p38 MAPK pathway that are secreted into the intestinal lumen [26]. The extracellular stage of N. parisii is the spore form, which has a tough coat that could be resistant to antimicrobials active in the lumen.

We also examined the *daf-2/daf-16* insulin/IGF signaling pathway for its role in resistance to N. parisii infection. While the *daf-2/daf-16* pathway did not appear to affect the rate of infection by N. parisii, it did affect the survival of infected animals. *daf-2* insulin receptor mutants were modestly resistant to infection, and this required the downstream transcription factor *daf-16*. It may be that in *daf-2* mutants, hyperactivation of *daf-16* induces expression of genes that are able to restrict the damage caused by N. parisii infection and thus allow animals to survive longer. DAF-16 controls expression of hundreds of genes, including antimicrobials and cellular stress response genes [33]. However, because *daf-2* mutants are generally long-lived, it is difficult to say whether the effect of *daf-2* is truly due to pathogen resistance, or to a more general effect on viability.

Despite the widespread use of C. elegans as a model organism for many biological processes including innate immunity, little is known about the natural ecology of this animal. C. elegans can be found in the soil, often on rotting fruit, and is thought to undergo a “boom and bust” lifestyle [34]. When food becomes available population levels rapidly increase, and when those food sources are exhausted, population levels crash and animals arrest in varying larval stages, which are able to withstand harsh conditions for long periods of time. In this context, N. parisii is particularly well suited to persist. During times of high population density, N. parisii can rapidly be transmitted from animal to animal. Once a N. parisii infection has been established, the infection can persist in arrested larvae (Figure 8A and unpublished data) to survive through times of less food and lower C. elegans population levels. N. parisii appears to be a widespread parasite of *Caenorhabditis* nematodes, as we found multiple natural isolates in France from widely separated localities that are infected with N. parisii as determined by rDNA sequence analysis. It will be interesting to examine the specificity of interaction between host/parasite pairs from different geographical regions, as well as the defense pathways used against these pathogens.

The phylogenetic analyses we performed on N. parisii and the related *Nematocida* species from C. briggsae indicate that they are unequivocally members of the microsporidia. The *Nematocida* lineage is closely related to O. popilliae, and tentatively to a group comprising *Paranosema* and *Antonospora*, which are members of Clade II [29]. The sister relationship between O. popilliae and the *Paranosema*+*Antonospora* group was first suggested by Vossbrinck and Andreadis [17] and our analysis suggests that *Ovavesicula*+*Nematocida* is sister to *Paranosema*+*Antonospora*. Interestingly, Vossbrinck and Andreadis [17] point out that O. popilliae, *Paranosema*, and *Antonospora* are all pathogens of terrestrial insects and that this group likely represents an independent origin of insect parasitism within the microsporidia (the other insect-infecting clade includes *Nosema* and *Vairimorpha* species within Clade IV of Vossbrinck and Debrunner-Vossbrinck [28]). Our analyses suggest that species in this group also attack terrestrial nematodes in the genus *Caenorhabditis.* It is noteworthy that *Nematocida* and *Ovavesicula* are both found in soil-dwelling invertebrates.

One intriguing feature of N. parisii microsporidian infection is that nematodes can sustain an impressive microbial load—virtually all of the intestinal cells can be filled—while still moving and appearing grossly normal. A recent study of malaria infection in mice suggested that animals tend to evolve either resistance (the ability to limit parasite burden) or tolerance (the ability to limit the damage caused by a given parasite burden) [35], a concept that has also been studied in plants [36,37] and *Drosophila* [38,39]. It seems likely that C. elegans has evolved a strategy of tolerance in response to microsporidian infection. For example, in lieu of destroying intestinal cells to exit the host, N. parisii infection appears to cause a subtle restructuring of the intestinal cells. Pathogens often evolve to cause only specific damage in order to minimize their impact on the host [40,41]. Evolutionary pressure for pathogen and host to coexist is likely to be especially strong in the case of obligate intracellular pathogens, which are completely dependent on their host to survive and replicate.

The restructuring of the terminal web caused by N. parisii infection in C. elegans may also occur in mammalian microsporidian infections, but has gone unrecognized because pathogens were assumed to exit only through the regular shedding of intestinal cells. Perhaps other intracellular pathogens of the intestine use a similar strategy to manipulate the intestinal cytoskeleton and then exit host cells. Several pathogens have been shown to manipulate the host cytoskeleton: for example *Listeria* has been shown to polymerize host actin in order to propel itself through the cell and infect neighboring cells [5]. However, little is known about pathogen interactions with intermediate filaments, which may be the target of N. parisii infection. There is evidence that other microsporidian infections may alter the structure of host cytoskeletal networks, perhaps via microsporidia-expressed intermediate filaments [42].

In addition to providing insight into the ecological pressures on the nematode C. elegans, the discovery of the microsporidian N. parisii may provide practical applications for agricultural pests. Parasitic nematodes are responsible for significant damage to multiple crop plants around the world and there is a need for more environmentally friendly management strategies [43,44]. Perhaps N. parisii could be used as a biocontrol agent to limit the spread of such parasitic nematodes. Microsporidia have already been used successfully as biocontrol agents for insects: in particular, the microsporidian *A. locustae,* which is in the same clade as N. parisii (Figure 2), is sprayed onto agricultural fields to control grasshoppers (Orthoptera) [45,46].

The discovery of N. parisii infection of C. elegans also provides a relatively inexpensive whole animal system in which to develop treatments for microsporidian infections in humans. Microsporidia are increasingly appreciated to be a serious medical problem. For example, there are no treatments available for infection by the species Enterocytozoon bieneusi, which is responsible for most of the microsporidian infections in humans [47]. Because microsporidia are obligate intracellular pathogens, screening for antimicrosporidia drugs requires the host to be present. The C. elegans/microsporidia model we have developed is an important advance in this regard. C. elegans are tiny (1-mm-long) hosts that can be used in high throughput screens. High throughput screens for antimicrobial compounds using C. elegans as a host have recently been developed in our laboratory and could be adapted to screen for molecules that prevent or cure microsporidian infection [48].

## Materials and Methods

### C. elegans isolation and strains.

Nematodes were isolated as described [49]. Briefly, compost or rotting fruit samples were placed onto a C. elegans culture dish around an Escherichia coli OP50 lawn and individuals were isolated as they came out of the sample. Some individuals were immediately observed by Nomarski optics (Figure 6A–6D).

The original N. parisii-infected strain, CPA24, was isolated in 2004 from a compost pile in Franconville, which is about 15 km north/northwest of Paris, France. The JU1247, JU1248, and JU1256 strains were established from infected individuals (a L2d larva, an egg-laying defective adult and a L2d larva, respectively) isolated from a rotting apple sampled on October 14, 2007 in a natural regional park in Santeuil, which is 50 km north/northwest of Paris. The population of *Caenorhabditis* nematodes in this apple was proliferating (non-dauer stages) and contained many C. elegans individuals and at least one C. briggsae individual that were infected by microsporidia: out of a total of 17 animals isolated, at least eight were infected based on examination by Nomarski (three more died without being examined).

Similar infections were also observed in two proliferating C. elegans populations from a Ficus isophlebia and an unidentified fruit in the Botanical Garden in Lisbon, Portugal (July 2005) and in C. elegans from mushroom compost outside a farm in Montsoreau, Maine-et-Loire, France (300 km south of Paris; March 2008). Strain JU1395 was established from an infected Montsoreau isolate.

Out of a total of 14 locations sampled from 2006–2008, infected C. elegans were found in four locations (Franconville, France; Lisbon, Portugal; Santeuil, France; and Montsoreau, France). Infected C. elegans were not found in the other ten locations (Hermanville, France; Le Blanc, France; Le Perreux, France; Primel-Sainte-Barbe, France; Merlet, France; Kakegawa, Japan; Concepcion, Chile; Sevilla, Spain; Carmona, Spain; Barcelona, Spain).

JU1348 is a microsporidian-infected C. briggsae strain established from a sample obtained in the Periyar Natural Preserve in Kerala, India. This area was one of eight areas sampled in Kerala, India where a *Caenorhabditis* species was found; infected animals were not found in the other seven areas*.* (Uninfected C. briggsae and other uninfected *Caenorhabditis* isolates were found in Estuary Island, Poovar; Botanical Garden, Trivandrum; near Meenmutti Waterfalls; Ponmudi Natural Preserve; Plantation near Kanjirapalli; Angela Spice Garden near Periyar. Uninfected C. brenneri were found in Allepey).

### rDNA isolation.

rDNA was either isolated from pure cultures of bacteria as described below, or directly from infected worms. To isolate bacterial 16S rDNA from infected worms, one to two infected worms were placed in a PCR tube with single egg/worm lysis buffer (SEWLB) (10 mM Tris-HCl [pH 8.3], 50 mM KCl, 2.5 mM MgCl2, 0.045% NP40, 0.045% Tween 20, 20 ng/μl proteinase K), and washed six to eight times with SEWLB over the course of an hour. Then, worms were lysed with a proteinase K treatment at 65 °C for 30 min to 1 h, followed by 15 min at 95 °C to inactivate the proteinase K. This extract was then subject to PCR using universal 16S primers. Bacterial 16S rDNA was isolated with the following primer sets:

8F (AGAGTTTGATCCTGGCTCAG) and 1492R (GGTTACCTTGTTACGACTT) [50]; 16SUF (CCGAATTCGTCGACAACAGAGTTTGATCCTGGCTCAG) and 16SUR (CCCGGGATCCAAGCTTACGGCTACCTTGTTACGACTT) [51]; 9FA (GAGTTTGATCITIGCTCAG) and 1513R (TACIGITACCTTGTTACGACTT) (Jacob Russell, personal correspondence); N331F(TCCTACGGGAGGCAGCAGT) and N797R (GGACTACCAGGGTATCTAATCCTGTT) [52]; 337F (CTCCTACGGGAGGCAGCAG) and 1100R (AGGGTTGCGCTCGTTG) [53].

With these primers, rDNA sequence was isolated that corresponded to an *Ochrobactrum* sp*.* and P. putida, which were isolated as described below.

For isolation of microsporidian rDNA, infected worms either were treated as above or were disrupted with silicon beads, the extract was filtered through Whatman filter paper number 1 and then subjected to PCR. Microsporidian sequence was isolated with 530F (GTGCCAGCMGCCGCGG) [53] and 1391R (GACGGGCGGTGWGTRCA) [54]. Further sequence was obtained with Micro308F (CCGGAGARGGAGCCTGAGA), which was designed based on alignment of other microsporidia species, and Micro648R (CGGTTCCGCACGGGCATC), which is specific to N. parisii, to obtain a 1,424-bp contig. A buffer-only PCR was always performed in parallel as a negative control, and samples were only used for cloning and sequencing if the negative control gave no signal by gel electrophoresis. PCR products were cloned into the TOPO TA vector (Invitrogen) and inserts were sequenced using primers that flank the insert of the TA vector. Sequences were analyzed by BLAST (and by the Ribosomal Database Project to find their closest match if they were bacterial).

### Bacterial strain isolation.

Infected worms were washed with M9 buffer several times, and then disrupted by vortexing with silicon beads. Extract was plated on several types of rich media, including tryptic soy agar, nutrient agar, and brain heart infusion agar. For universal 16S PCR 1 μl of overnight culture was mixed with 4 μl of SEWLB buffer, incubated at 65 °C for 30 to 60 min, then 95 °C for 15 min to inactivate the protease. This lysate was then used in PCR reactions. Pure cultures of an *Ochrobactrum* sp*.* and P. putida were obtained with this method. Feeding these bacteria to uninfected worms, either alone, in combination, or in a variety of dilutions did not confer the intracellular infection. The P. putida infection was lost through regular propagation of infected worms on E. coli strain OP50, however, Ochrobactrum sp. could only be removed from infected worms by repeatedly incubating worms in buffer containing the antibiotic gentamicin (15 μg/ml) for 1–2 h at a time over several generations (see Text S1 for further information on *Ochrobactrum*).

### FISH staining on infected worms.

C. elegans intestines were dissected as described [55]. Briefly, adults were transferred to a drop of M9 with levamisole on a microscope slide and the heads and tails were cut with a 25-gauge needle to extract the intestines. Samples were transferred to a microfuge tube and fixed with 4% paraformaldehyde for 1–2 h. FISH was then performed essentially as described for bacteria [56]. Samples were washed with PBS + 0.1% Tween 20 and then transferred to hybridization buffer (900 mM NaCl, 20 mM Tris [pH 7.5], 0.01% SDS) containing 5 ng/μl probe. Probes were designed against regions of the ribosomal sequence specific to N. parisii and were synthesized with a Quasar 570 (Cy3) 5′ modification and HPLC purified by Biosearch Technologies, Inc. Two N. parisii-specific probes were tested and gave similar results: MicroA (CTCTGTCCATCCTCGGCAA) and MicroB (CTCTCGGCACTCCTTCCTG). These probes also cross-react with the Nematocida sp. 1 sequence isolated from JU1348, the infected C. briggsae strain from India. The universal bacterial probe EUB338 (GCTGCCTCCCGTAGGAGT) was synthesized the same way. Hybridization was performed at 46 °C overnight. Intestines were washed at 48 °C for 1 h in wash buffer (900 mM NaCl, 20 mM Tris [pH 7.5], 0.01% SDS, 5 mM EDTA) and then mounted for microscopy with Vectashield containing DAPI (Vector Laboratories). For Figure 3A–3C, staining was performed in parallel and exposure times were the same for all. FISH staining was also performed as above, except without intestine dissection and with acetone fixation for 15 min at room temperature [57] instead of paraformaldehyde fixation. These conditions allowed for better staining of spores and were used for the FISH staining shown in Figure 3E and Figure 8G–8I.

### Phylogenetic placement of *Nematocida.*

In order to place *Nematocida* in phylogenetic context small subunit ribosomal RNA sequences from 57 placeholder taxa (downloaded from GenBank) were chosen from each of the major clades of microsporidia based on the phylogenetic hypotheses of Vossbrinck and Debrunner-Vossbrinck [29]. This included two fungi (Basidiobolus ranarum and Conidobolus coronatus) *sensu* Vossbrinck and Debrunner-Vossbrinck [28] as outgroups, which is consistent with the hypothesis that the Microsporidia's closest living relatives are fungi. We included the N. parisii sequence and the *Nematocida* sequence (*Nematocida* sp1.) isolated from Indian C. briggsae strain JU1348. The small subunit ribosomal RNA sequences were aligned using the default parameters in the Clustal W2 web interface (http://www.ebi.ac.uk/Tools/clustalw2/index.html) [58], and the alignment was visually inspected (the alignment is available upon request). We trimmed all unalignable insertions and deletions from the entire alignment. The resulting alignment comprised 940 characters and 59 taxa. We then used the three mostly widely used phylogenetic methods (Bayesian inference, maximum likelihood, and parsimony) to place the *Nematocida* accessions phylogenetically.

### Parsimony.

To evaluate nodal support for evolutionary relationships among the 59 taxa, we performed a parsimony bootstrap heuristic search in Paup (phylogenetic analysis using parsimony [and other methods], version 4.0b10; Swofford, D. L. 4.0 Beta. Sinauer Associates, Inc.) [59], with 1,000 replicates using the 59 taxa alignment. Of the 940 total characters, 255 were constant, 69 variable characters were parsimony uninformative, and 616 were parsimony informative. Gaps were treated as missing data. Starting trees were obtained via stepwise addition, with simple sequence addition (reference taxon was Encephalitozoon lacerate), the branch-swapping algorithm was tree-bisection-reconnection and the steepest descent option was not in effect. A bootstrap consensus tree was computed from this heuristic search.

### Maximum likelihood.

We determined the most likely model of nucleotide substitution across these sequences using the Modeltest program, version 3.71 [60]. The TrN+I+G model was chosen as the most likely (−lnL = 18,797). We then used the PhyML [61] algorithm through the PhyML 3.0 webserver (http://www.atgc-montpellier.fr/phyml/). This program is based on the algorithm of Guindon and Gascuel [62] and constructs an initial tree using distance methods and then performs a phylogenetic search using the maximum likelihood optimality criterion. It is especially useful with large datasets. We chose the GTR model of nucleotide substitution (the TrN+I+G model is derived from the GTR model) in PhyML and performed 100 bootstrap replicates on the dataset to determine support values for relationships in the phylogeny (on the single most-likely tree, −lnL = 18,727).

### Bayesian inference.

We used the program MrModeltest 2.2 [63] to estimate the most likely model of sequence evolution (MrBayes utilizes a subset of the models used by Paup) prior to estimating the phylogeny using Bayesian inference. The GTR+I+G model of nucleotide substitution was chosen as the most likely (−lnL = 32,299). We then used the program MrBayes 3.1.2 [64] to estimate the phylogeny using Bayesian inference, which also yields posterior probabilities of nodes in the phylogeny using a Markov chain Monte Carlo (MCMC) approach. The analysis was performed using two independent runs, with four chains each (temp = 0.2), and each chain (sampled every 100) was run for ten million generations. After completion of the run, we examined convergence rates of posterior split probabilities in the MCMC using the program AWTY [65]. Convergence was obtained after ∼6 million generations. A consensus tree for each run was obtained that gave average branch lengths and posterior probability values; the two consensus trees were topologically identical.

In accordance with section 8.6 of the ICZN's International Code of Zoological Nomenclature, we have deposited copies of this article at the following five publicly accessible libraries: National Museum of Natural History, Smithsonian Institute, Washington (D.C.), United States of America; Museum of Comparative Zoology, Harvard University, Cambridge, Massachusetts, United States of America; Museum National d'Histoire naturelle, Paris, France; California Academy of Sciences, San Francisco, California, United States of America; San Diego Natural History Museum, San Diego, California, United States of America. The new genus and species names established herein have been registered in ZooBank [66], the official online registration system for the ICZN. The ZooBank publication LSID (Life Science Identifier) for the new species described herein can be viewed through any standard web browser by appending the LSID to the prefix http://zoobank.org/.

### TEM.

C. elegans were fixed in 2.5% glutaraldehyde, 1.0% paraformaldehyde in 0.05 M sodium cacodylate buffer, (pH 7.4) plus 3.0% sucrose. The cuticles were nicked with a razor blade in a drop of fixative under a dissecting microscope to allow the fixative to penetrate. After 1 h fixation at room temperature, the worms were fixed overnight at 4 °C. After several rinses in 0.1 M cacodylate buffer, the samples were postfixed in 1.0% osmium tetroxide in 0.1 M cacodylate buffer for 1 h at room temperature. They were rinsed in buffer and then in double distilled water and stained, en bloc in 2.0% aqueous uranyl acetate for 1 h at room temperature (for lighter staining of mature spores, this first uranyl acetate staining step was sometimes omitted). After rinsing in distilled water, the last rinse was carefully drawn off and the worms were embedded in 2.0% agarose in PBS for ease of handling. The agarose blocks were dehydrated through a graded series of ethanol to 100%, then into 100% propylene oxide, and finally into a 1:1 mixture of propylene oxide:EPON overnight on a rocker. The following day, the agarose blocks were further infiltrated in 100% EPON for several hours and then were embedded in fresh EPON overnight at 60 °C. Thin sections were cut on a Reichert Ultracut E ultramicrotome and collected on formvar-coated gold grids. They were poststained with uranyl acetate and lead citrate and viewed in a JEOL 1011 TEM at 80 kV equipped with an AMT digital imaging system (Advanced Microscopy Techniques).

### Measuring spore size.

To measure spore size by light microscopy, infected nematodes containing spores were photographed using Nomarski optics on a Zeiss AxioImager microscope and then spores were measured using the measurement function on Zeiss Axiovision software. Values given are the average ±SD. To measure spore size by TEM, sections of infected nematodes were photographed and then measured using AMT version 5 software.

### Pharyngeal pumping assays.

Infected or noninfected animals were placed individually on NGM plates seeded with OP50 and allowed to acclimate for 10–30 min. Videos of pharyngeal pumping were then recorded of each animal for 15–60 s using a Sony Handycam attached to a Zeiss M2 microscope. Videos were analyzed using iMovie and pumps were counted using the slow motion feature. Rates described are the average ±SD and are from two separate experiments.

### Antibody staining.

C. elegans intestines were dissected as described [55]. Briefly, adults were transferred to a drop of M9 with levamisole on a microscope slide and the heads and tails were cut with a 25-gauge needle to extract the intestines. Samples were transferred to a microfuge tube with M9 + 0.1% Tween 20 to prevent sticking, and fixed with 4% paraformaldehyde for 1–2 h. Samples were washed three times with PBS 0.1% Tw20 and then incubated in block for 1–2 h. Block was PBS, 0.5% TX100, 1 mM EDTA, 0.1% BSA, 0.05% sodium azide, adjusted to pH 7.2 with hydrochloric acid. Next, samples were incubated in the primary antibody MH33 (Hybridoma Bank), 1:100 in block, overnight at 4 °C. Samples were then washed four times in PBS, then incubated in the secondary antibody Cy3-labeled goat anti-mouse IgG (Jackson Immunoresearch), 1:1,000 in block at room temperature for 2 h. Samples were washed three times in PBS, then mounted in Vectashield with DAPI for viewing by fluorescence microscopy.

### N. parisii infection assays.

Infectious N. parisii extract was made as follows. Infected worms were washed with M9 several times over the course of 1 h in a 2-ml microfuge tube. Silicon carbide beads (BioSpec Products, Inc.) were added to the tube, and the tube was vortexed for 1 min, four to five times. The worm extract was then filtered through Whatman filter paper number 1 to remove eggs and any remaining intact worms, glycerol was added to a final concentration of 15%, and aliquots were frozen at −80 °C. Extracts were tested for contamination by plating onto TSA bacterial media plates. To begin an infection assay, aliquots were thawed on ice, and then 50 μl of extract (usually diluted 1:5 or 1:10) was added to a lawn of OP50 seeded on a 6-cm NGM plate. Synchronized L1s or L4/young adults were then added to these plates. For survival assays animals were transferred to new plates approximately 2 d after becoming adults and then every day afterward while they were producing progeny, in order to prevent progeny from obscuring the assay.

Time to 50% of animals exhibiting symptoms was analyzed with Prism software using nonlinear regression analysis with the error described as standard error of the mean. For meronts, vesicles, and survival curves in Figure 5A, a Boltzmann sigmoidal provided the best fit. A Boltzmann sigmoidal curve did not provide a good curve fit for spore appearance (Boltzmann sigmoidal fit calculated an aberrantly long time to 50% of animals with spores), so a sigmoidal curve was used, although this does not provide a perfect curve fit. In order to determine when there was a statistically significant difference between infected and uninfected animals as described in the legend of Figure 5, an unpaired two-tailed *t*-test in Excel software was used to compare these two populations.

Strains were compared using the same batch of infectious N. parisii in parallel, with three to four plates tested per strain, per experiment. Each strain was analyzed in at least three independent experiments. For each timepoint 120 to 150 worms of each strain (40 to 50 worms from each plate) were mounted on agarose pads and scored by DIC microscopy for the presence of grooves, rod-shaped microbes, or vesicles of microbes. Animals were tested for survival by prodding with a platinum wire. N2, *pmk-1(km25), daf-2(e1368ts), daf-2(e1368);daf-16(mgDf47)* strains were raised at 20 °C and then shifted to 25 °C for assays. In experiments including *daf-2* mutants, strains were transferred to 25 °C (the restrictive temperature for *daf-2*) several hours prior to inoculation. Statistical analysis of symptoms in different strains was performed with an unpaired two-tailed *t*-test in Excel software. Statistical analysis of survival of different strains was performed with log-rank analysis in Prism software.

### Testing infected donor animals for ability to transmit infection to recipients.

For initial characterization of horizontal transmission, single adult donor animals of a marked genotype (e.g., Dpy) were placed on plates with several adult recipient animals, and then recipient animals were examined for the presence of infection several days later. For transmission analysis graphed in Figure 5B, a single infected donor adult animal was co-incubated on the same plate as 200–300 L1 recipient animals for 12 h, then removed and examined by DIC microscopy for the presence of meronts or spores. 2–3 d later recipients (*n* > 30 animals) were examined by DIC microscopy for signs of infection.

### Quantitative reverse transcriptase-PCR analysis.

Animals were grown until the L3/L4 stage, and then transferred to OP50-seeded NGM plates with or without N. parisii. Animals were harvested 34 h later, because this was when the first signs of infection (grooves) were apparent in the majority of animals under these conditions. For P. aeruginosa and S. aureus infections, animals were treated as described (J.I. Irazoqui, E.R. Troemel, F.M. Ausubel, unpublished data) [26,27]. Total RNA was then extracted using TRI Reagent, and reverse transcribed using the Retroscript kit (Ambion). This cDNA was then subjected to quantitative reverse transcriptase (qRT)-PCR analysis using SYBR green detection on an iCycler machine (BioRad). Primers for qRT-PCR were designed using Primer3 (MIT), checked for specificity against the C. elegans genome, and tested for efficiency with a dilution series of template. All values are normalized against *nhr-23*, which is a control gene that does not vary under these conditions. Fold difference was calculated using the Pfaffl method. Statistical significance was assessed by a one-sample *t*-test. Primer sequences are available upon request.

### Accession numbers.

Small subunit ribosomal RNA sequences have been uploaded to Genbank for N. parisii (FJ005051), *Nematocida* sp. 1 (FJ005052), and *Ochrobactrum* sp. (FJ005053).

## Supporting Information

Figure S1 *Ochrobactrum*-GFP Remains Restricted to the Intestinal Lumen until Death (865 KB PDF)

Figure S2 Quantitative RT-PCR Analysis of Animals Infected with N. parisii
(598 KB PDF)

Text S1 Results, Discussion, Materials and Methods, and References for Isolation and Characterization of Novel *Ochrobactrum* Species (61 KB DOC)

## Abbreviations

DAPI: 4′,6-diamidino-2-phenylindole
DIC: differential interference contrast
FISH: fluorescence in situ hybridization
GFP: green fluorescent protein
IGF: insulin-like growth factor
L: larval stage
MAPK: mitogen-activated protein kinase
TEM: transmission electron microscopy
