# Supplementary material for: Microsporidia Are Natural Intracellular Parasites of the Nematode Caenorhabditis elegans
Source: PLoS Biol. 2008 Dec 9;6(12):e309. doi: 10.1371/journal.pbio.0060309 (PMC2596862; doi:10.1371/journal.pbio.0060309)
Supplement: Supplementary file 1 [file pbio.0060309.sg001.pdf]

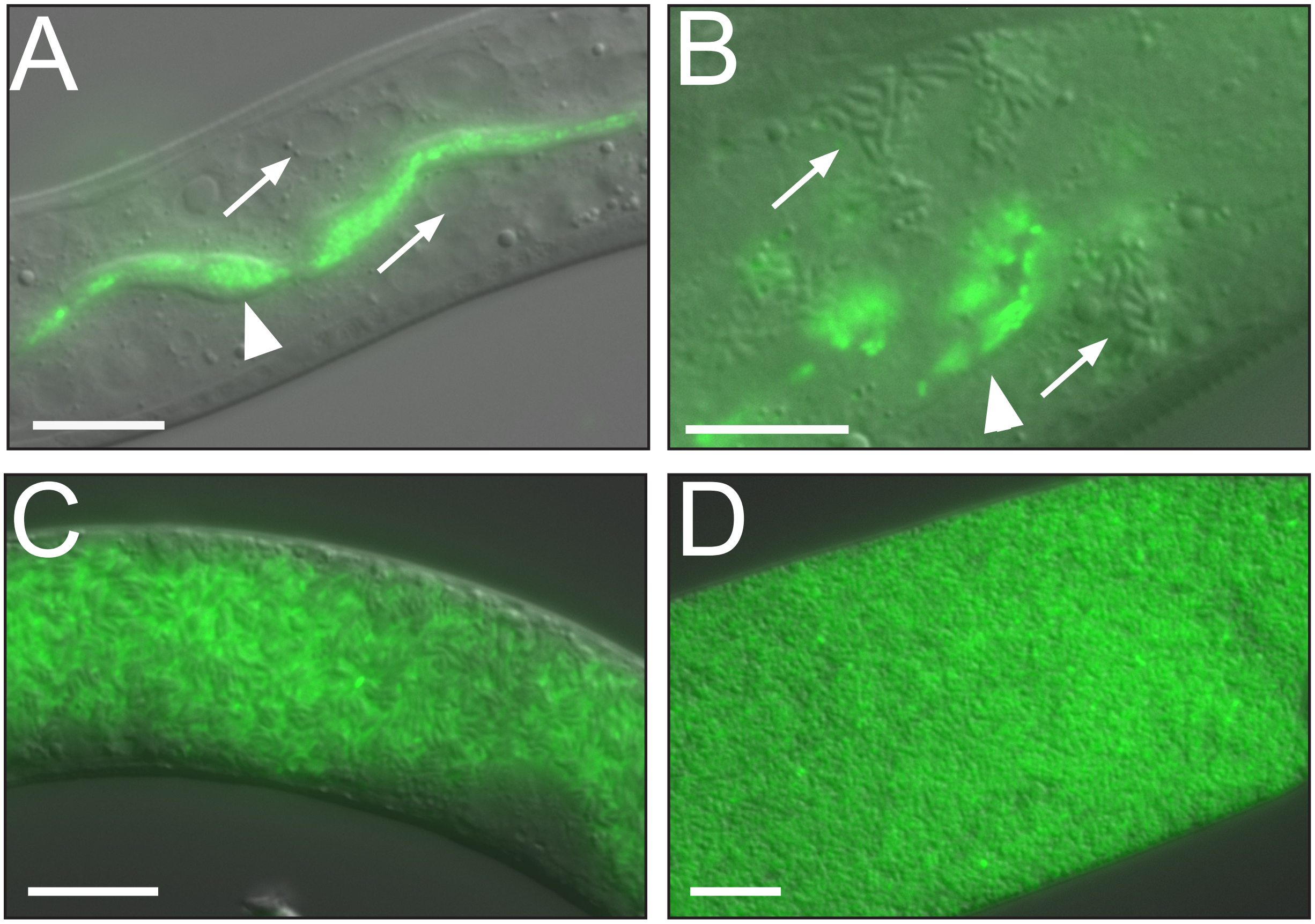
A
B
C
D
