# Supplementary material for: Microsporidia Are Natural Intracellular Parasites of the Nematode Caenorhabditis elegans
Source: PLoS Biol. 2008 Dec 9;6(12):e309. doi: 10.1371/journal.pbio.0060309 (PMC2596862; doi:10.1371/journal.pbio.0060309)
Supplement: Supplementary file 2 [file pbio.0060309.sg002.pdf]

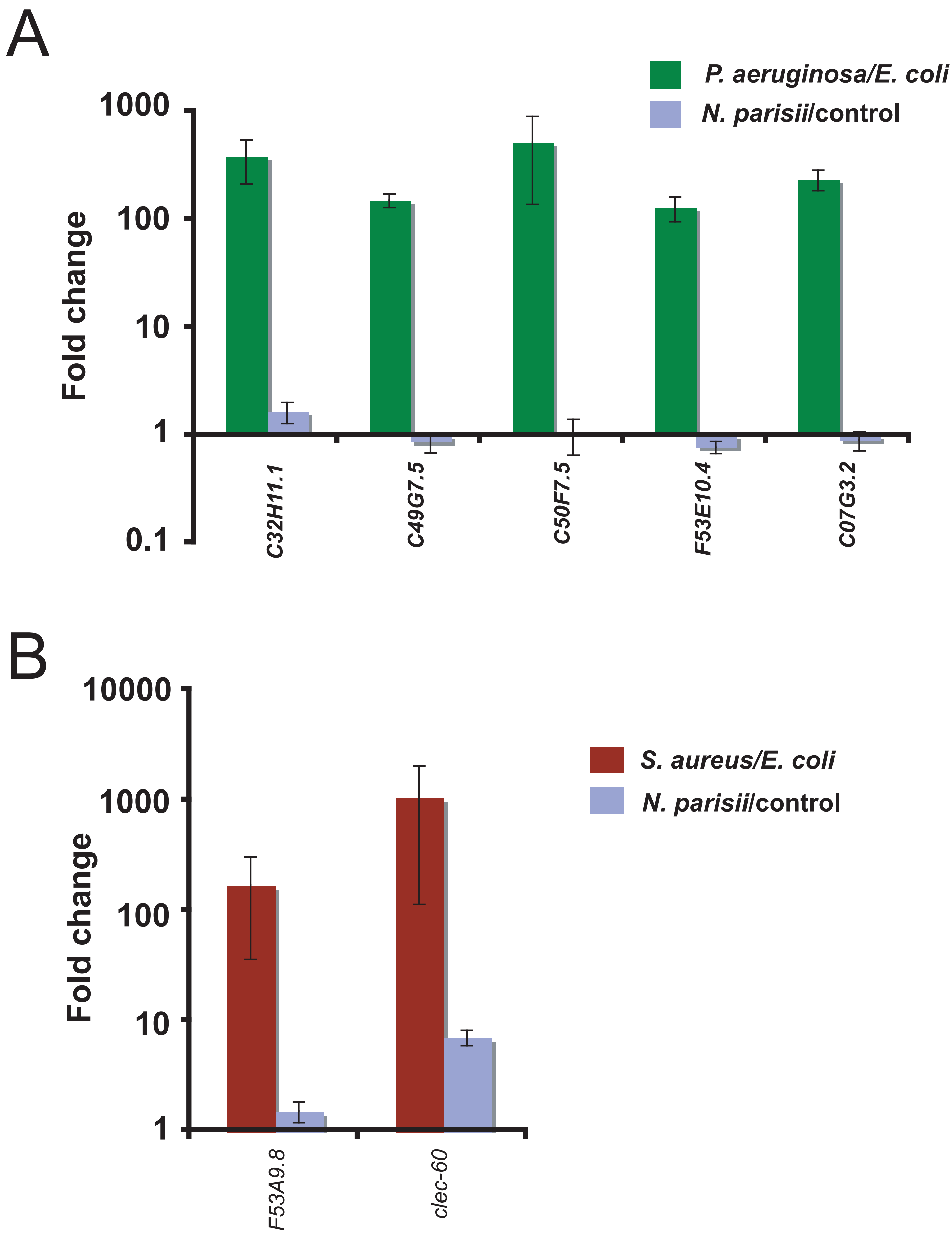

A
P. aeruginosa/E. coli
N. parisii/control
1000
100
10
1
0.1
Fold change
C32H11.1
C49G7.5
C50F7.5
F53E10.4
C07G3.2
B
10000
1000
100
10
1
Fold change
S. aureus/E. coli
N. parisii/control
F53A9.8
clec-60
