# Supplementary material for: Microsporidia Are Natural Intracellular Parasites of the Nematode Caenorhabditis elegans
Source: PLoS Biol. 2008 Dec 9;6(12):e309. doi: 10.1371/journal.pbio.0060309 (PMC2596862; doi:10.1371/journal.pbio.0060309)
Supplement: Supplementary file 3 [file pbio.0060309.sd001.doc]

**Supplemental Results**

In addition to microsporidia, we also isolated a new strain of the Gram negative bacterium, *Ochrobactrum*, from *N. parisii*-infected animals. Localizing GFP-labeled *Ochrobactrum* in infected worms indicated that it remained restricted to the intestinal lumen until animals had died, at which point it proliferated throughout the animal (Figure S1).

**Supplemental Discussion**

*Ochrobactrum* is a bacterium commonly found in the soil: in one study of French soil, it comprised about 2% of all bacterial strains isolated [1]. Because this strain of *Ochrobactrum* remained associated with the microsporidian-infected strain of worms for over 18 months of regular strain propagation on *E. coli* (data not shown), it apparently forms a fairly tight association with *C. elegans*. GFP-labeled *Ochrobactrum* remained restricted to the lumen in microsporidian-infected animals until death of the host, but they then spread throughout animals at that time (Figure S1). *Ochrobactrum* has also been isolated from entomopathogenic nematodes [2], suggesting that *Ochrobactrum* may commonly be associated with nematodes.

### Supplemental Materials and Methods

### Labeling *Ochrobactrum* with GFP by triparental mating

In order to label *Ochrobactrum* in the context of infected worms with intracellular microbes, infected worms were fed two *E. coli* strains: the helper mating strain MT616, and a strain carrying pHC60, which is a plasmid that drives constitutive expression of GFP [3]. After 48 hours, infected worms were disrupted and the extract was plated on media containing kanamcyin to select for the *Ochrobactrum* sp. (which is kanamycin-resistant) and against the *E. coli* strains. The media also contained tetracycline, which selects for the presence of the GFP plasmid. Under these conditions, only GFP-labeled *Ochrobactrum* colonies grew. Previous experiments had shown that animals fed on plates (that only contained these *Ochrobactrum* colonies by light microscopy and 16S rDNA PCR typing) became infected with the intracellular infection. Therefore, uninfected animals were fed on these GFP-labeled *Ochrobactrum* colonies, which presumably also contained *N. parisii*, and they became infected with intracellular microbes. Upon inspecting infected worms however, GFP-labeled *Ochrobactrum* was restricted to the lumen of infected worms until death, in which case it was found throughout the corpse (Figure S1).

**Supplemental References**

1. Bathe S, Achouak W, Hartmann A, Heulin T, Schloter M, et al. (2006) Genetic and phenotypic microdiversity of Ochrobactrum spp. FEMS Microbiol Ecol 56: 272-280.

2. Babic I, Fischer-Le Saux M, Giraud E, Boemare N (2000) Occurrence of natural dixenic associations between the symbiont Photorhabdus luminescens and bacteria related to Ochrobactrum spp. in tropical entomopathogenic Heterorhabditis spp. (Nematoda, Rhabditida). Microbiology 146 ( Pt 3): 709-718.

3. Cheng HP, Walker GC (1998) Succinoglycan is required for initiation and elongation of infection threads during nodulation of alfalfa by Rhizobium meliloti. J Bacteriol 180: 5183-5191.

### Supplemental Figure Legends

### Figure S1. *Ochrobactrum*-GFP remains restricted to the intestinal lumen until death.

(A) Animal infected with *Ochrobactrum*-GFP at the groove stage of intracellular infection. Arrows indicate grooves, arrowhead indicates *Ochrobactrum*-GFP restricted to the intestinal lumen. (B) Animal infected with *Ochrobactrum*-GFP at the rod-shaped microbe stage of intracellular infection, restricted to the intestine. Arrows indicate rod-shaped microbes not labeled with GFP, arrowhead indicates *Ochrobactrum*-GFP restricted to the lumen. (C) Animal recently dead from intracellular infection has *Ochrobactrum*-GFP spreading into intestine. (D) *Ochrobactrum*-GFP is found throughout an extensively degraded corpse. For all panels, scale bar is 10 µm.

### Figure S2. Quantitative RT-PCR analysis of animals infected with *N. parisii*.

(A) Comparison of gene induction by *P. aeruginosa* and *N. parisii*. (B) Comparison of gene induction by *S. aureus* and *N. parisii*. Fold change is calculated for *P. aeruginosa* by comparing expression in animals feeding on *P. aeruginosa* to animals feeding on *E. coli* OP50, for *S. aureus*, by comparing expression in animals feeding on *S. aureus* to animals feeding on *E. coli* OP50 and for *N. parisii* by comparing animals feeding on *N. parisii* and *E. coli* to animals feeding on just *E. coli*. For *P. aeruginosa* and *N. parisii*, fold changes are the average of three biological replicates and for *S. aureus*, fold changes are the average of two biological replicates. Each biological replicate was measured in duplicate and normalized to a control gene. Error bars are SEM. *N. parisii* did not significantly induce any of these genes except *clec-60* (p=0.03).
